# Acetaminophen Does Not Reduce Postoperative Opiate Consumption in Patients Undergoing Craniotomy for Cerebral Revascularization: A Randomized Control Trial

**DOI:** 10.7759/cureus.3863

**Published:** 2019-01-10

**Authors:** Mark A Burbridge, Sarah A Stone, Richard A Jaffe

**Affiliations:** 1 Anesthesiology, Stanford University Medical Center, Stanford, USA

**Keywords:** acetaminophen, post-operative pain, craniotomy, moyamoya disease, postoperative pain

## Abstract

Background

Postoperative management in patients undergoing craniotomy is unique and challenging. We utilized a population of patients who underwent bilateral extracranial-to-intracranial (EC-IC bypass) revascularization procedures for moyamoya disease and hypothesized that 1 gram (gm) of intravenous (IV) acetaminophen given immediately after intubation and again 45 minutes prior to the end of craniotomy may be more effective than saline in minimizing opiate consumption and decreasing pain scores.

Methods

In a double-blind, randomized, placebo-controlled crossover pilot study, 40 craniotomies in 20 patients were studied. A random number generator assigned patients to receive either 1 gram of IV acetaminophen or an equal volume of normal saline immediately after intubation and again 45 minutes prior to the end of their first operation. For the second surgery, patients received the study drug (IV acetaminophen or normal saline) that they did not receive during their first surgery.

Results

In the IV acetaminophen group, the average 24-hour postoperative fentanyl equivalent consumption was decreased but the difference was not statistically significant: 228 micrograms compared to 312 micrograms in the placebo group (Figure 1; p = 0.09). Pain scores did not significantly differ between the IV acetaminophen group and the placebo group in postoperative hours 0-12 (Figure 2; p = 0.44) or 24 (Figure 3; p = 0.77).

Conclusion

Our study demonstrates that in patients receiving bilateral craniotomies for moyamoya disease, IV acetaminophen when given immediately after intubation and again 45 minutes prior to closure does not significantly decrease 12- or 24-hour postoperative opiate consumption.

## Introduction

Postoperative management in patients undergoing craniotomy is unique and challenging. An ideal anesthetic would dissipate rapidly, permitting the patient to participate in a postoperative neurologic exam to identify any new deficits while still in the operating room. Accordingly, practitioners may minimize opioid use with the result that post-craniotomy pain is often undertreated [1-3]. In addition to unwanted sedation, opioid use may also be limited due to side effects, including hypoventilation and the resultant hypercapnia, postoperative nausea and vomiting, and the increased risk of delirium.

Alternate modalities for postoperative pain control after craniotomy have been explored, including non-steroidal anti-inflammatory drugs (NSAIDs), dexmedetomidine, gamma-aminobutyric acid (GABA) analogs/gabapentinoids (e.g. gabapentin), acetaminophen and supplemental regional anesthesia. NSAIDs have been shown to decrease postoperative analgesic requirements, however, they may impair surgical hemostasis and contribute to postoperative bleeding [4]. Dexmedetomidine and GABA analogs can cause excessive sedation. Dexmedetomidine can also cause undesired hemodynamic effects, including bradycardia and hypotension.

Acetaminophen is a well-tolerated and effective analgesic that can be used in an intravenous (IV) formulation perioperatively, to help manage pain. The mechanism of action of acetaminophen is largely undetermined; however, it is thought to function through central processes, including effects on prostaglandin production, serotonergic, opioid, nitric oxide (NO), and cannabinoid pathways [5]. The intravenous formulation possesses several important differences as compared to the oral or rectal routes of administration and results in a faster time to peak plasma drug concentration (15 minutes after initiation of infusion as compared to two and three hours for oral and rectal administration, respectively) and peak plasma levels that are up to three times greater [6].

While studies have shown that a single dose of intravenous (IV) acetaminophen given once intraoperatively [4], and re-dosed at regular intervals postoperatively [7-8] does not produce adequate analgesia, we hypothesized that 1 gm IV acetaminophen given immediately after intubation and again 45 minutes prior to the end of craniotomy may be more effective in minimizing opiate consumption and decreasing pain scores in the 24-hour postoperative period.

We utilized a unique population of patients who underwent bilateral extracranial-to-intracranial (EC-IC bypass) revascularization procedures for moyamoya disease (ICD-10: I67.5). Each patient underwent two identical surgeries and, therefore, was able to serve as their own control. This unique experimental design and the use of two doses of IV acetaminophen is a novel approach in assessing the effectiveness of IV acetaminophen for postoperative pain after craniotomy.

## Materials and methods

Patients

This is a double-blind, randomized, placebo-controlled pilot study, approved by the Institutional Research Board of Stanford University. Forty craniotomies in 20 patients were studied. Inclusion criteria for this study included patients aged over 18 years, patients presenting for bilateral EC-IC bypass revascularization surgery for moyamoya disease, and no preoperative or postoperative language deficits. Exclusion criteria were pre-existing hepatic dysfunction, a history of substance abuse, allergy, or intolerance to acetaminophen or any associated excipients, patient refusal, chronic pain, pregnancy, and previous craniotomy.

Anesthesia

All patients included in the study were given a standard anesthetic regimen for both surgeries: Pre-medication with midazolam 1-2 milligrams (mg) in the preoperative holding area was given at the discretion of the anesthesiologist for anxiolysis. At the time of induction, fentanyl 7-9 micrograms (mcg)/kilogram (kg) was administered in divided doses, followed by 0.5 mg/kg propofol and 0.6 mg/kg rocuronium. Patients then underwent endotracheal intubation. Dexamethasone 8 mg IV was given pre-incision to reduce surgical swelling and for postoperative nausea and vomiting (PONV) prophylaxis. Ceftriaxone 1 gm IV or cefazolin 2 gm IV was given for surgical site infection prophylaxis. Patients were maintained on a mixture of 50% nitrous oxide, 50% oxygen, 0.5 age-adjusted minimum alveolar concentration (MAC) isoflurane or sevoflurane, and a remifentanil infusion of 0.05 - 0.2 mcg/kg/minute (min) titrated to obtain hemodynamic stability. Cerebral perfusion pressure was maintained by keeping mean arterial pressure between 80 and 110 mmHg. The adequacy was often assessed using electroencephalographic and evoked potential techniques. Patients who were determined by history to be at higher risk for PONV received propofol 50 mcg/kg/min as a substitute for nitrous oxide. All patients were monitored with invasive arterial blood pressure monitoring. Patients who underwent combined direct-indirect EC-IC bypass (n= 16) received a central venous catheter and were cooled by way of a water circulation blanket to achieve a target temperature of 33 degrees Celsius at the time of cross-clamping of an M4 branch of the middle cerebral artery (MCA). Such patients were re-warmed with a target temperature of > 35 degrees Celsius at the time of extubation. Patients undergoing only an indirect EC-IC bypass did not receive central venous catheters or cooling. All patients received ondansetron 8 mg and metoclopramide 10 mg 30 minutes prior to extubation. All patients were extubated and evaluated in the operating room after discontinuing the remifentanil infusion and then transferred to the intensive care unit for subsequent monitoring.

Study protocol

A random number generator was used by the hospital pharmacy to assign patients to receive either 1 gm of IV acetaminophen or an equal volume (100 milliliters) of normal saline immediately after intubation and again 45 minutes prior to the end of their first operation. For the second surgery, patients received the study drug (IV acetaminophen or normal saline) that they did not receive during their first surgery, both immediately after intubation and again 45 minutes prior to the end of the procedure. The average length of surgery was 396.5 minutes. Each patient served as their own control.

Anesthesiologists were not blinded to which study drug the patient received, nor were they involved in postoperative pain assessment or treatment. Patients were managed in an identical manner intraoperatively, according to the aforementioned study protocol. All patients were extubated at the conclusion of the surgery and transferred to the intensive care unit (ICU) in the same manner. Upon arrival to the ICU, an information packet was given to the bedside nurse and attending physician, both of whom were blinded to the intervention that had been given in the operating room. Routine nursing assessments were conducted per protocol by the nursing team for the next 24 hours. Each patient’s opioid consumption, pain scores, and antiemetic consumption were monitored and recorded in the electronic medical record. In addition to the study drug, a standard analgesic regimen, consisting of fentanyl, hydrocodone, and hydromorphone, was administered to all patients at the discretion of the ICU medical and nursing teams.

Outcomes measured

Primary outcomes for the 40 craniotomies in 20 patients included in this study were postoperative opioid requirements and postoperative pain scores over the first 24 hours. The secondary outcome was antiemetic use.

## Results

Patients

The majority of our study patients were female (16 female, four male), and the average age was 37.8 years. The average time between surgeries was 11 days (minimum seven days, maximum 42 days). Sixteen patients received bilateral combined direct-indirect EC-IC surgery. Three patients received bilateral indirect EC-IC surgery. One patient received both indirect and direct EC-IC surgeries.

Postoperative opioid consumption

**Figure 1:**
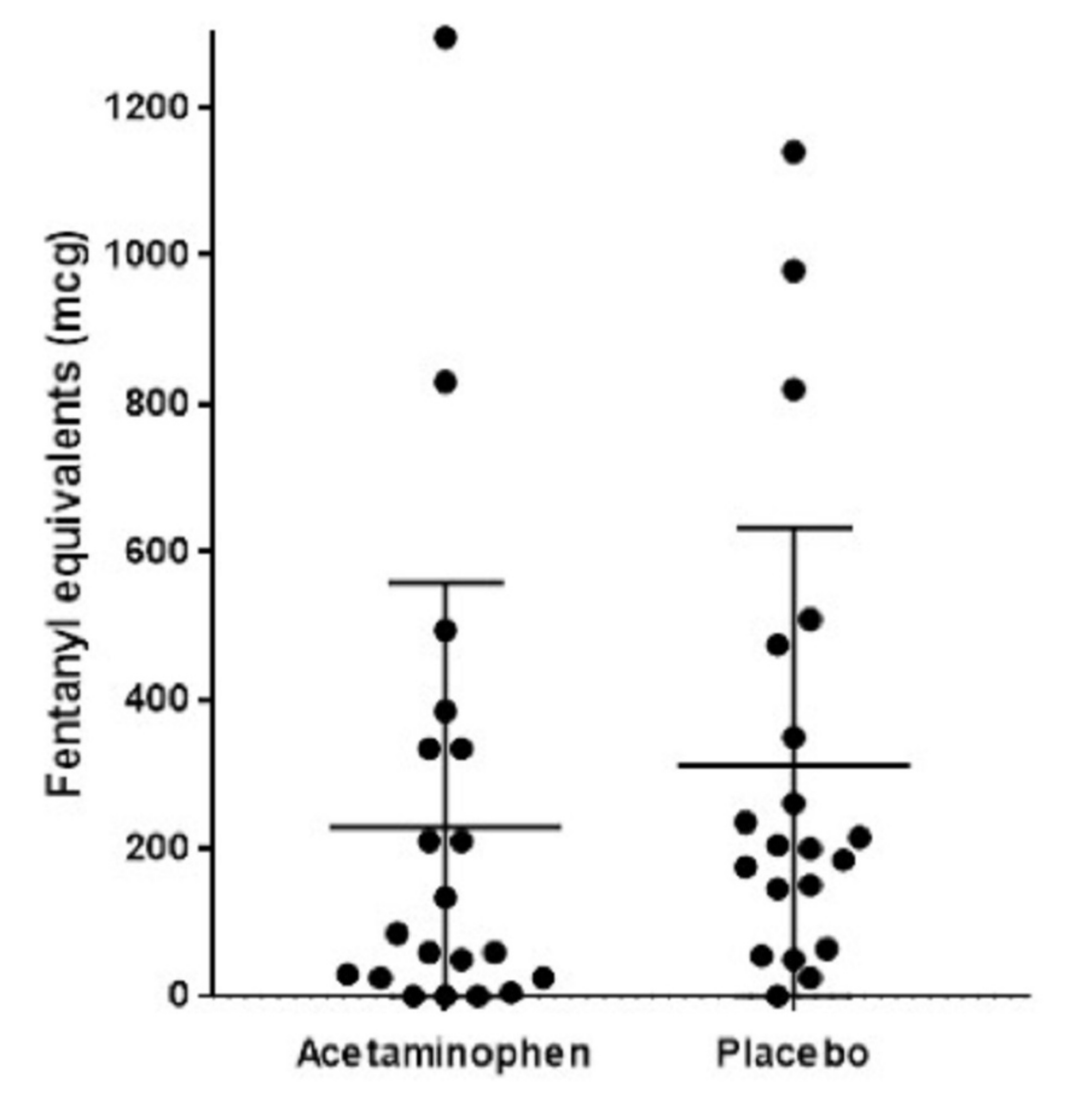
Scatter plot demonstrates decreased average 24-hour postoperative fentanyl consumption (measured in fentanyl equivalents) without statistical significance, 228 micrograms as compared to 312 micrograms in the placebo group (p = 0.09)

In the IV acetaminophen group, the average 24-hour postoperative fentanyl equivalent consumption was decreased but the difference was not statistically significant: 228 mcg as compared to 312 mcg in the placebo group (Figure 1; p = 0.09). When divided into postoperative hours 0-12, fentanyl use in the IV acetaminophen group was 162 mcg as compared to 224 mcg in the placebo group (p = 0.1). In postoperative hours 13-24, fentanyl use in the IV acetaminophen group was 67 micrograms as compared to 88 micrograms in the placebo group (p = 0.3).

Pain scores

**Figure 2:**
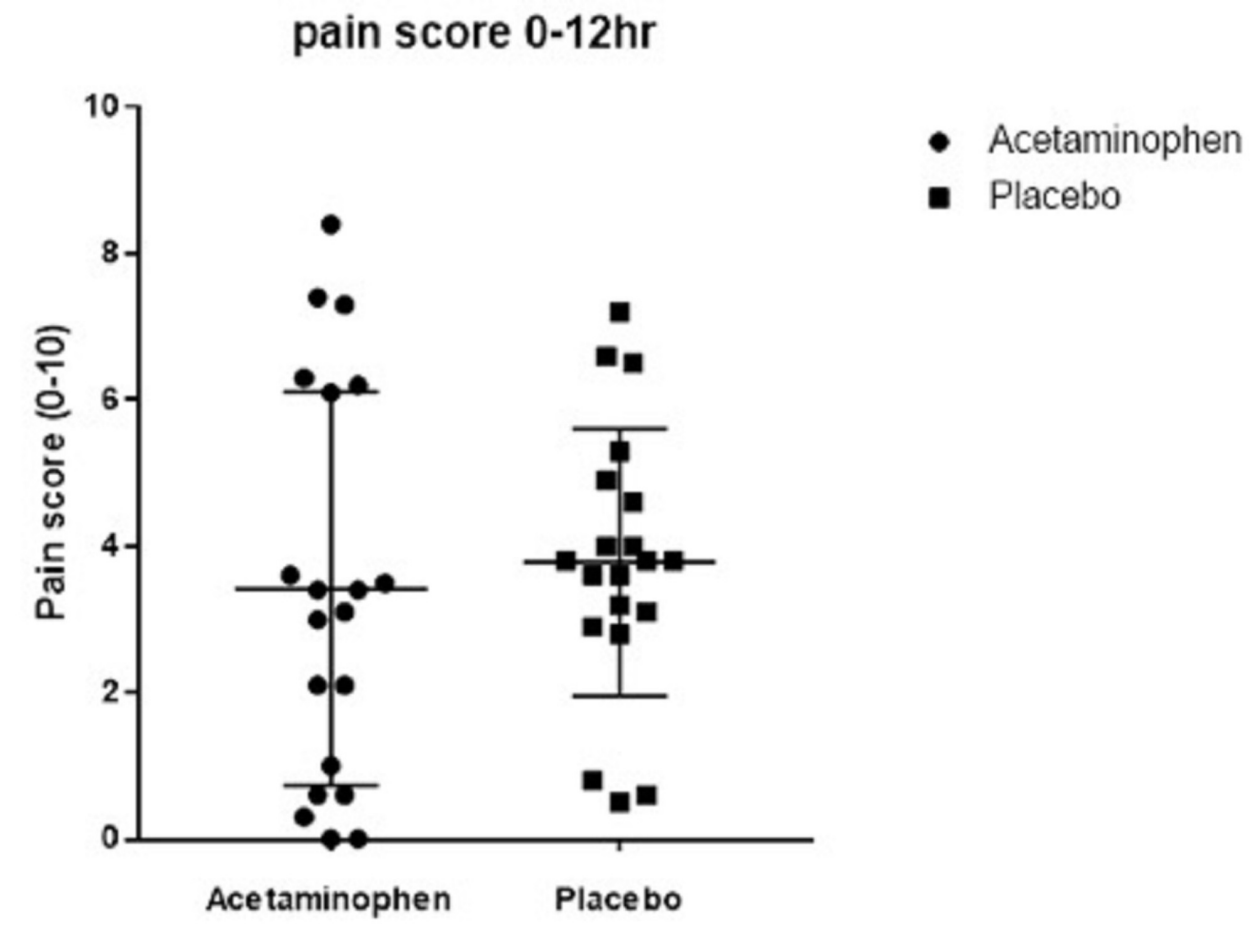
Scatter plot demonstrates that pain scores, measured by Visual Analogue Scale (0-10), did not significantly differ between the IV acetaminophen group and the placebo group in postoperative hours 0-12 (p = 0.44) IV: intravenous

**Figure 3:**
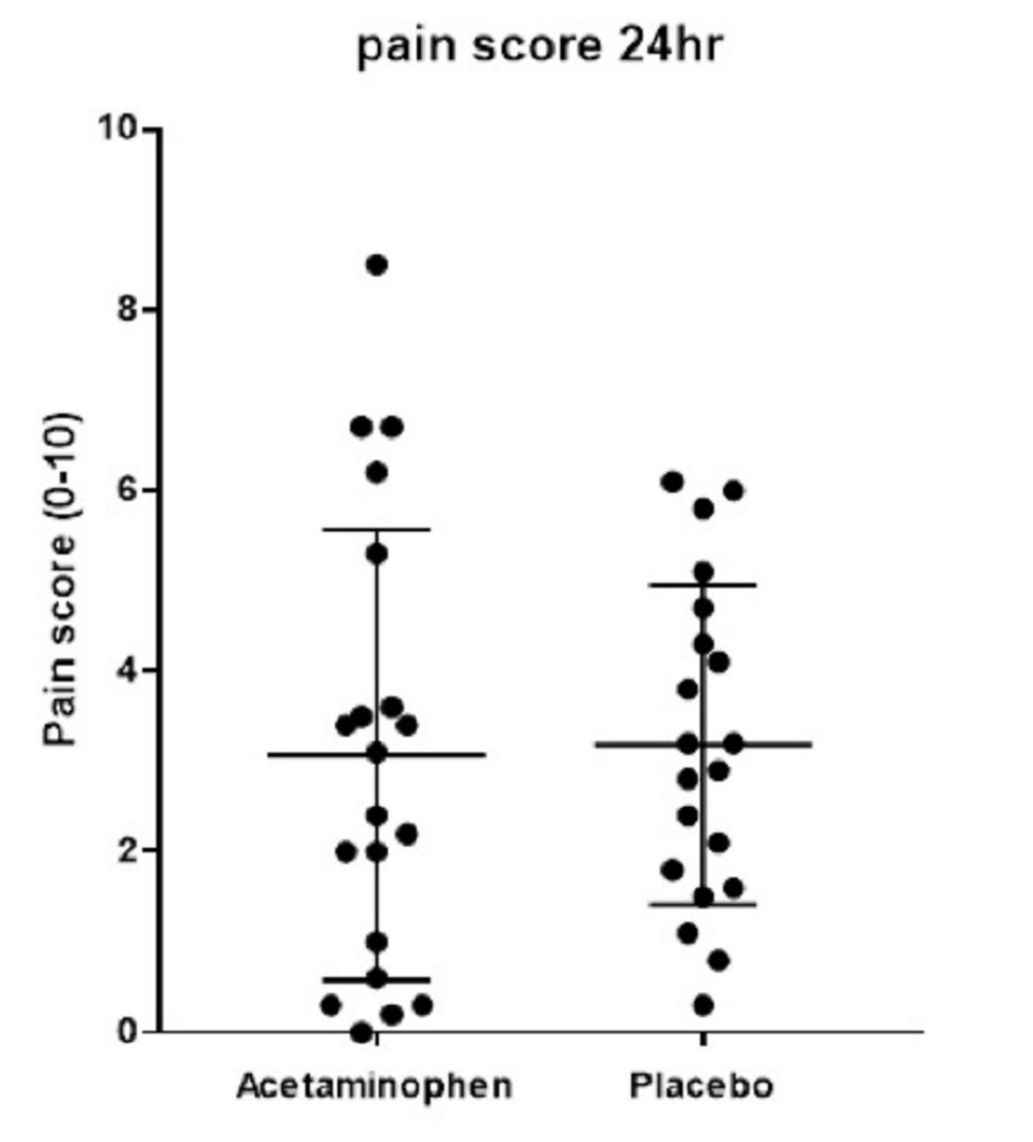
Scatter plot demonstrates average pain scores over 24 hours (p = 0.77) measured by the Visual Analogue Scale (0-10)

Pain scores did not significantly differ between the IV acetaminophen group and the placebo group in postoperative hours 0-12 (Figure 2; p = 0.44) or 24 (Figure 3; p = 0.77).

Antiemetic use

**Figure 4:**
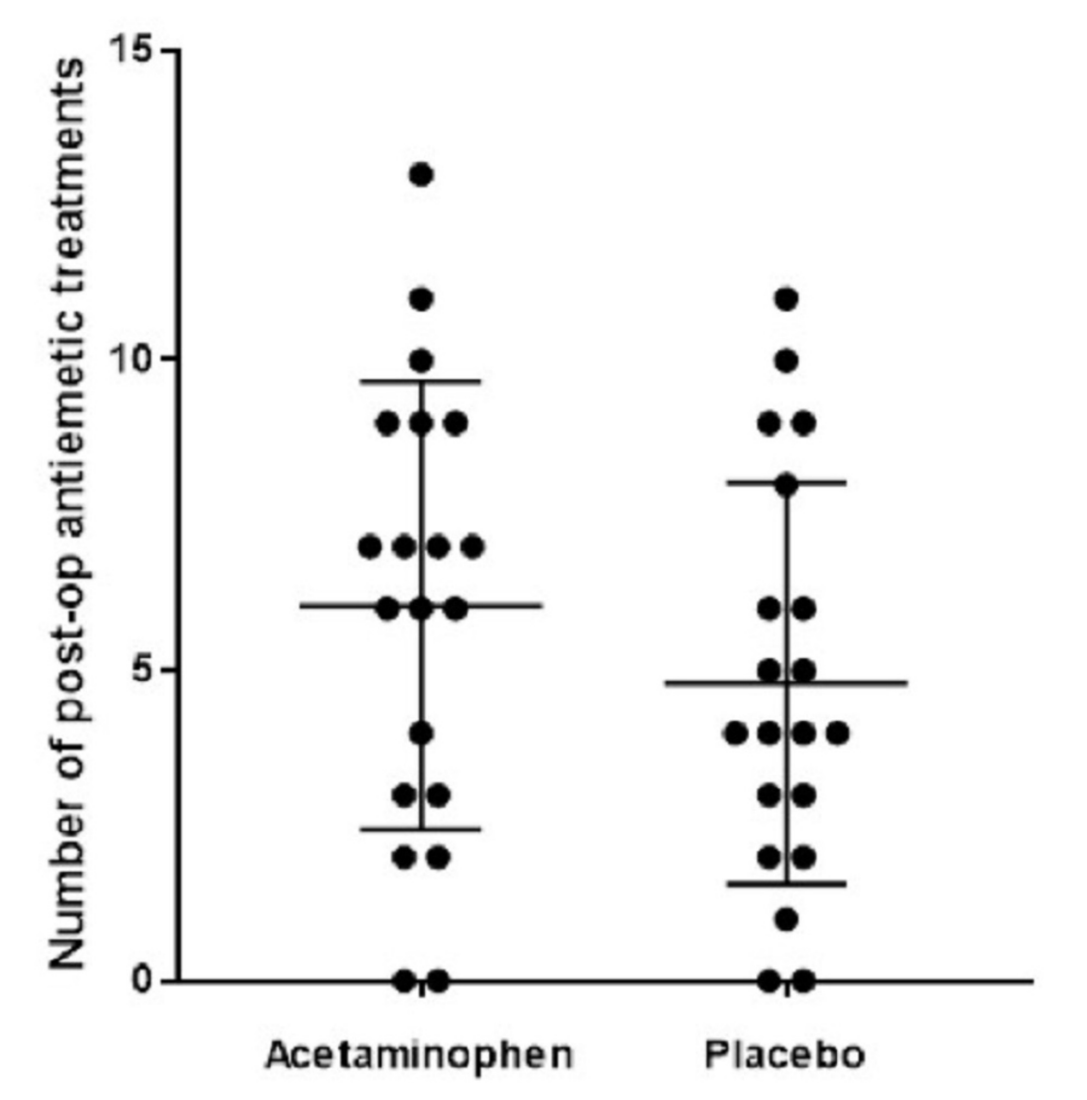
Scatter plot demonstrates no statistically significant difference in 24-hour postoperative antiemetic dosage between the IV acetaminophen and placebo groups (2-tailed matched t-test, p = 0.19) IV: intravenous

There was no statistically significant difference in the 24-hour, postoperative antiemetic dosage between the IV acetaminophen and placebo groups (Figure 4; 2-tailed matched t-test, p = 0.19). One dose of antiemetic was defined as ondansetron 4 mg, metoclopramide 10 mg, and granisetron 1000 mcg.

## Discussion

Moyamoya disease is a rare cerebrovascular disease, characterized by progressive stenosis of the distal internal carotid arteries and proximal anterior and middle cerebral arteries. This flow restriction results in the formation of abnormal vascular collaterals in the ischemic territory [9]. Patients can present with symptoms of transient ischemic attacks, strokes, headaches, and seizures. There is no effective medical therapy, and surgery is necessary to bypass the blocked arteries and restore normal blood flow. The surgical treatment of bilateral moyamoya disease involves bilateral sequential craniotomies with direct anastomosis of an M4 branch of the MCA to a branch of the superficial temporal artery (STA) (direct bypass) or the simple placement of the STA branch in contact with the cortical surface (indirect bypass). Our study makes use of a unique neurosurgical population: the same patient receives the same surgery on each side of the brain, approximately one to two weeks apart, and, therefore, can serve as their own control, randomized to receive either acetaminophen or placebo on the first side, with the opposite treatment at the time of their second surgery.

Opioid consumption

Our data demonstrate that IV acetaminophen, when given immediately after intubation and again 45 minutes prior to closure, does not significantly decrease postoperative opioid consumption at 12 or 24 hours. Our data parallel several recent studies, including one by Dilmen et al. in 2016, which demonstrated that supplemental analgesics (dexketoprofen and metamizole) did not affect post-craniotomy morphine consumption [3], and a study by Artime et al. in 2017, which demonstrated that IV acetaminophen did not show an opioid-sparing effect after craniotomy but was associated with improved patient satisfaction regarding overall pain control [10]. Interestingly, several studies in emergency departments compared IV acetaminophen to morphine for the treatment of acute pain and demonstrated no statistical difference in pain scores, analgesic effect, or rescue analgesic administration [11-13], highlighting the analgesic effect of acetaminophen. It is important to note, however, that the effects of acetaminophen on postoperative opioid consumption may be more difficult to detect when the average pain score is lower. 

Given the data presented above and the practice of anesthesia in today’s cost-conscious environment, providers might be discouraged from utilizing IV acetaminophen as an adjunct to treat post-craniotomy pain. However, given that IV acetaminophen has been shown to improve patient satisfaction after craniotomy and has been shown to be equally effective as morphine in acute pain, providers may consider utilizing oral or rectal formulations of acetaminophen as part of a multimodal treatment approach.

There are a number of limitations to our study, including that it was underpowered: a post hoc power analysis indicated an achieved power of 0.4 and suggested that an n of 50 would be needed to detect a statistically significant acetaminophen effect during the first 12 hours after surgery. Unfortunately, there were no studies upon which to base a pre-trial power analysis, but we felt that if our results were clinically significant, an n of 20 would be adequate to demonstrate this. A second limitation was that several patients within our cohort requested medications from their home regimen that were not included in our ICU protocol, including benzodiazepines for anxiety (e.g. lorazepam), and pain adjuncts, including the combination drug acetaminophen/butalbital/caffeine. Lastly, of the medications that were included in the ICU postoperative pain protocol, a sliding scale of IV and oral opiate medications were available for the nursing staff to administer and there may have been variation among nursing staff in how much of the PRN (or as needed) medications were given.

Clinical studies such as this one present unique challenges in that it is impossible to control for all patient and environmental variables. The intention of our study was to assess the utility of IV acetaminophen in a real-world setting, with the hope that if acetaminophen had a significant effect in postoperative opioid consumption, the results would be apparent despite an n of 20.

Pain scores

Our data demonstrate that there was no statistically significant difference in the 12- or 24-hour postoperative pain scores between groups. Pain scores were similar between groups, suggesting that patients were treated similarly between interventions, indicating the adequate blinding of the postoperative ICU medical and nursing staff.

Antiemetic use

Increased opiate consumption increases a patient’s risk of undesired side effects, including nausea and vomiting. We hypothesized that decreased opiate consumption would be associated with decreased PONV and, consequently, the consumption of antiemetics. Our data demonstrate no statistically significant difference in antiemetic consumption in the 24-hour postoperative period between the IV acetaminophen and placebo groups. This is consistent with the lack of significance in postoperative opioid consumption discussed previously. This may be attributable to the nature of the surgery itself, as patients receiving craniotomy are known to be at higher risk for PONV [14]. Some data suggest there is an effect of acetaminophen to lower PONV that is independent of its opioid-sparing effect [15]. Several limitations in this aspect of our study exist. First, there is no established method to measure antiemetic medication equivalents between patients who receive different medication regimens for managing PONV. Second, differences exist in the nursing assessment of nausea and how much of the PRN antiemetic is administered. Third, nausea, like pain, is a subjective parameter, but unlike pain, it has no defined scale for measurement. A more rigorous protocol may have assisted in standardizing the treatment of PONV in this study.

## Conclusions

In conclusion, our study demonstrates that in patients receiving bilateral craniotomies for moyamoya disease, IV acetaminophen, when given immediately after intubation and again 45 minutes prior to closure, does not significantly decrease 12- or 24-hour postoperative opiate consumption, although the amount of opiate given was noted to be less in the IV acetaminophen group. There was no difference in the 24-hour postoperative pain scores or antiemetic usage. Despite our small sample size of 40 craniotomies in 20 patients, we believe our study answers a novel question in that a pre-emptive dose of IV acetaminophen given before incision, combined with a second dose given near the end of surgery, does not appear to add a significant opioid-sparing effect on postoperative pain control but may be a useful adjunct in the multimodal pain management strategy after craniotomy.

## References

[1] Stoneham MD, Walters FJ (2018). Post-operative analgesia for craniotomy patients: current attitudes among neuroanaesthetists. Eur J Anaesthesiol.

[2] Mordhorst C, Latz B, Kerz T (2010). Prospective assessment of postoperative pain after craniotomy. J Neurosurg Anesthesiol.

[3] Dilmen OK, Akcil EF, Tunali Y (2016). Postoperative analgesia for supratentorial craniotomy. Clin Neurol Neurosurg.

[4] Hoefnagel A, Lopez M, Mitchell K, Smith D, Feng C, Nadler J (2015). Intravenous acetaminophen administration in patients undergoing craniotomy - a retrospective institutional study. J Anesth Clin Res.

[5] Lachiewicz PF (2013). The role of intravenous acetaminophen in multimodal pain protocols for perioperative orthopedic patients. Orthopedics.

[6] Chiam E, Weinberg L, Bellomo R (2018). Paracetamol: a review with specific focus on the haemodynamic effects of intravenous administration. Heart Lung Vessel.

[7] Nair S, Rajshekhar V (2011). Evaluation of pain following supratentorial craniotomy. Br J Neurosurg.

[8] Verchère E, Grenier B, Mesli A, Siao D, Sesay M, Maurette P (2002). Postoperative pain management after supratentorial craniotomy. J Neurosurg Anesthesiol.

[9] Achrol AS, Guzman R, Lee M, Steinberg GK (2009). Pathophysiology and genetic factors in moyamoya disease. Neurosurg Focus.

[10] Artime CA, Aijazi H, Zhang H (2017). Scheduled intravenous acetaminophen improves patient satisfaction with postcraniotomy pain management. J Neurosurg Anesthesiol.

[11] Bektas F, Eken C, Karadenız O, Goksu E, Cubuk M, Cete Y (2009). Intravenous paracetamol or morphine for the treatment of renal Colic: a randomized, placebo-controlled trial. Ann Emerg Med.

[12] Craig M, Jeavons R, Probert J, Benger J (2012). Randomised comparison of intravenous paracetamol and intravenous morphine for acute traumatic limb pain in the emergency department. Emerg Med J.

[13] Serinken M, Eken C, Turkcuer I, Elicabuk H, Uyanik E, Schultz CH (2012). Intravenous paracetamol versus morphine for renal colic in the emergency department: a randomised double-blind controlled trial. Emerg Med J.

[14] Latz B, Mordhorst C, Kerz T (2011). Postoperative nausea and vomiting in patients after craniotomy: incidence and risk factors. J Neurosurg.

[15] Apfel CC, Turan A, Souza K, Pergolizzi J, Hornuss C (2013). Intravenous acetaminophen reduces postoperative nausea and vomiting: a systematic review and meta-analysis. Pain.

